# Supplementary material for: Controlled ultrafast ππ*-πσ* dynamics in tryptophan-based peptides with tailored micro-environment
Source: Commun Chem. 2021 Aug 31;4:124. doi: 10.1038/s42004-021-00557-4 (PMC9814788; doi:10.1038/s42004-021-00557-4)
Supplement: Supplementary file 1 — Supplementary Information [file 42004_2021_557_MOESM1_ESM.pdf]

Supplementary Information: Controlled Ultrafast ππ*-πσ* Dynamics in Tryptophan-based Peptides with Tailored Micro-environment

M. Hervé, A. Boyer, R. Brédy, I. Compagnon, A.-R. Allouche and F. Lépine

Univ Lyon, Université Claude Bernard Lyon 1, CNRS, Institut Lumière Matière, F-69622, VILLEURBANNE, France

Supplementary Methods

1. Femtosecond Pump-Probe Experiments of On-the-fly Molecular Ions

Supplementary Figure 1 shows a comparison of the mass spectrum obtained for $TrpH^+$ with and without 266nm irradiation, together with the time-resolved mass spectrum for m/z=130 displayed in the main text (Figure 1b). As can be seen, the mass spectrum without irradiation already contains several fragments (m/z=188, 146,…), due to collisions with the residual gas in the spectrometer, matching typical CID fragments[1] of $TrpH^+$. When interaction with UV photons occurs, the yield of specific fragments is increased as observed in previous studies[2], for instance for m/z=130 or 132. However, no H-loss (m/z=204) was observed in our experiment, while it has been reported in the literature[2]. This is due to the residual gas present in the instrument that induces secondary fragmentation of any created m/z=204 fragment into m/z=130, which is more stable (the overall background pressure inside the instrument is about 10^{-5} mbar, except inside the collision cell (kept at an activation voltage of 0 V) with an argon pressure of about 4.10^{-3} mbar, necessary to guide the ions with a good transmission efficiency toward MS2). Supplementary Figure 1b shows the transient signal obtained for fragment m/z=130, corresponding to the ππ*-πσ* dynamics after UV excitation.

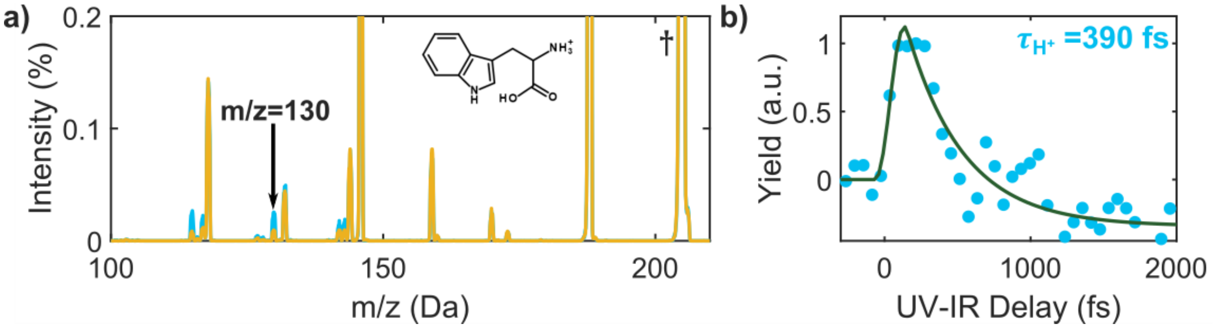

Supplementary Figure 1. Time-resolved mass spectrometry measurements in $TrpH^+$. a) Mass spectrum for $TrpH^+$ after interaction with 266nm photons (light blue spectrum), superimposed with the same spectrum without irradiation (yellow spectrum). The spectra are normalized on the parent signal (m/z=205), indicated by the † marker. b) Normalized time-dependent yield of fragment m/z=130, as a function of the UV pump-IR probe delay (blue dots), together with its exponential fit (green line).

Supplementary Figure 2 displays the time-dependent m/z=130 yield of $TrpNa^+$, recorded on the femtosecond timescale. While a lifetime of 13 ps is observed on the picosecond timescale, the short delay (0-2 ps) behaviour only reveals an apparent step evolution, corresponding to the early instants of the relaxation. In contrast, in the case of $TrpH^+$, excitation at 266nm showed the existence of 2 de-excitation timescales[2], of 380 fs and 15 ps, where the first one is attributed to the fast ππ*-πσ* coupling, and the long one corresponds to internal conversion down to the ground state, through excited-state

proton transfer, as proposed in [3]. These two mechanisms are known to compete at excitation energies above the origin of the ππ* band. In the case of $TrpNa^+$, the major presence of the photofragment m/z=130 reveals a charge motion that is intrinsic to ππ*-πσ* couplings. The observation of a single lifetime for $TrpNa^+$ directly confirms the attribution of the measured timescale to this coupling. As in the case of $TrpH^+$, the observed plateau after the picosecond dynamics in $TrpNa^+$ is also a signature of radical $Trp^{\bullet+}$ absorption, once the transfer dynamics is complete[4].

In both protonated and sodiated species, the observed timescale corresponds to the population transfer from the ππ* state to the πσ* state. Once the transfer is complete, dissociation occurs through different ways. While for $TrpH^+$ the creation of an unstable (hypervalent) R-NH_3 radical can lead to the m/z=130 observable by H-loss and subsequent radical fragmentation of $Trp^{\bullet+}$ or by other channels such as C-N dissociation[5], the dynamics in $TrpNa^+$ mostly results in the formation and loss of neutral Na, giving the $Trp^{\bullet+}$ radical and its subsequent fragmentation to m/z=130. Overall, the m/z=130 observable is a signature of the ππ*-πσ* dynamics.

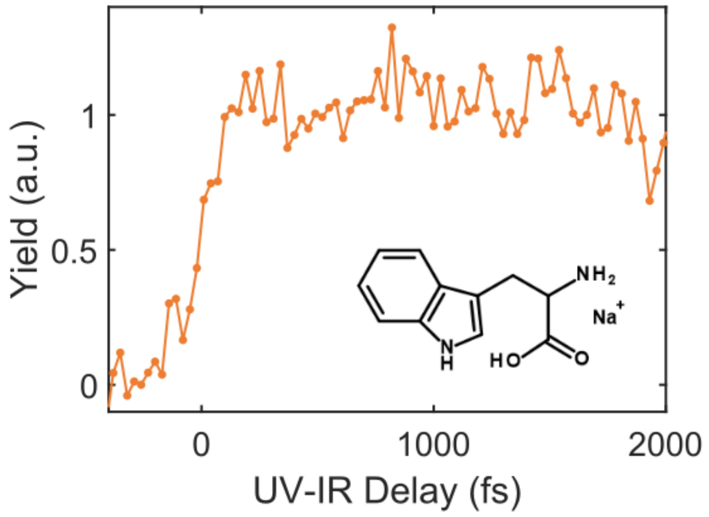

Supplementary Figure 2. Femtosecond dynamics in $TrpNa^+$. Normalized time-dependent yield of fragment m/z=130 for $TrpNa^+$, as a function of the UV-IR delay on a femtosecond timescale.

Finally, Supplementary Figure 3 shows the result of the interaction of UV photons with sodiated alanyl-tryptophan, $AlaTrpNa^+$. While residual fragmentation is observed without irradiation, different fragments are produced by UV absorption, with m/z=168, 170 and 187 as the main products. In this case, the fragment m/z=130 is not observed, because of the more complex structure of the dipeptide. The fragment m/z=187 however corresponds to $[M\text{-}Ala\text{-}Na]^+$, i.e. a loss of the Na atom and a cleavage of the tryptophan C_α-NH bond together with an hydrogen transfer. It thus corresponds to a relocalization of the charge on the indole moiety, as for m/z=130. Similarly, m/z=170 is attributed to $[M\text{-}Ala\text{-}Na\text{-}OH]^+$, while m/z=168 corresponds to the C_α-C_β cleavage of the tryptophan moiety with the charge carried by the alanyl moiety, as in the ground state of the molecule. These fragments indicate that, again, a charge motion occurs after UV excitation, that is characteristic of the ππ*-πσ* coupling mechanism.

The three fragments (m/z=168, 170 and 187) exhibit a similar dynamical behaviour, shown in Supplementary Figure 3b. Using a multidimensional fitting procedure, a single timescale of 35±8 ps is found for all the fragments. Again, special care was taken to investigate their dynamical behaviour on the femtosecond timescale (Supplementary Figure 3c), and a step evolution is observed as for $TrpNa^+$, confirming the absence of fast dynamics.

For all the species studied here ($TrpH^+$, $TrpNa^+$ and $AlaTrpNa^+$), the transient signals are shown in absolute value and normalized to their respective maximum, in order to compare the obtained dynamics

between the different molecular systems. In all cases, absorption of IR probe photons projects the ππ* population on higher-lying states, thus changing the relative fragmentation yields through an increase of the total energy.

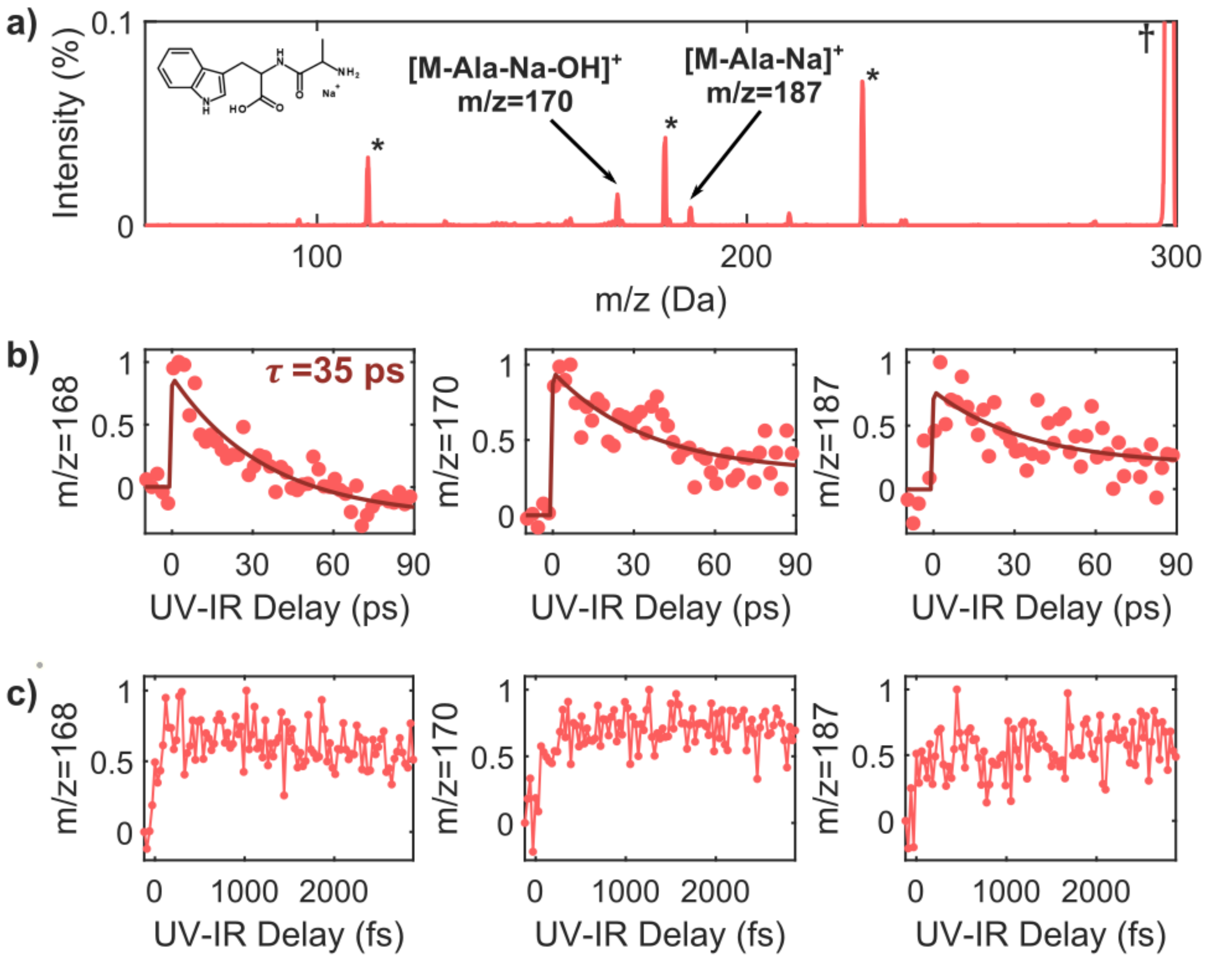

Supplementary Figure 3. Time-resolved mass spectrometry measurements in AlaTrpNa$^+$. a) Mass spectrum for AlaTrpNa$^+$ after interaction with 266nm photons, normalized on the parent signal (m/z=298), indicated by the † marker. The * markers indicate fragments that are due to residual fragmentation. b) Normalized time-dependent yields of the most abundant photofragments as a function of the UV-IR delay: m/z=168, 170 and 187. The multidimensional fit is shown in brown full line. c) Same signal as b), on a femtosecond timescale.

2. Ground State Geometry of Tryptophan Derivatives

As described in the Computational Methods section of the main text, geometries were optimized by DFT calculations using the B3LYP functional at the 6-311+G(d,p) level. Supplementary Figure 4 displays the geometry of the main conformers below 100 meV (9.6 kJ.mol^{-1}) for the three molecules considered in this work. For TrpH$^+$, 3 conformers are found. In particular, conformer 2 is close in energy from the optimized geometry, as already observed in previous studies[3]. The position of its excited states is however very similar to conformer 1, as shown later in Supplementary Table 1. In the case of TrpNa$^+$, only one conformer is found, and corresponds to N/O/ring chelation of the Na$^+$ adduct. Its geometry is shown in Supplementary Figure 5 in more details. Finally, 4 conformers below 100 meV exist for AlaTrpNa$^+$. Given their higher energy, conformers 2 to 4 are much less present within our experiment.

The gas phase geometry of AlaTrpNa$^+$ thus corresponds to a complex of Na$^+$ by the indole ring, the NH_2 moiety of the alanyl residue and the carbonyl group of the peptide bond.

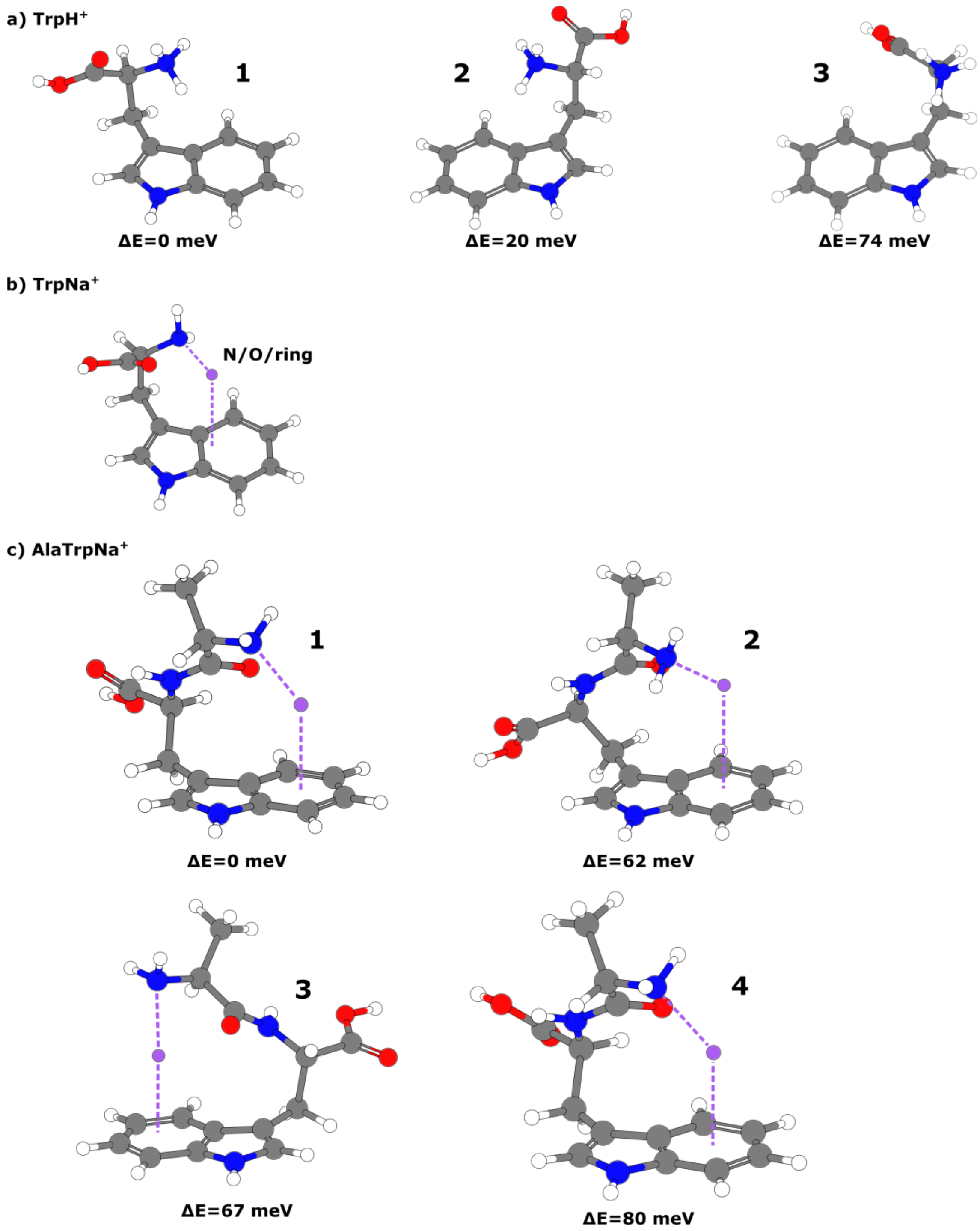

Supplementary Figure 4. Calculated geometries of the studied tryptophan-containing species. Conformation analysis of the different molecules considered in this study. All the conformers below 100 meV are shown for TrpH$^+$ (a), TrpNa$^+$ (b) and AlaTrpNa$^+$ (c).

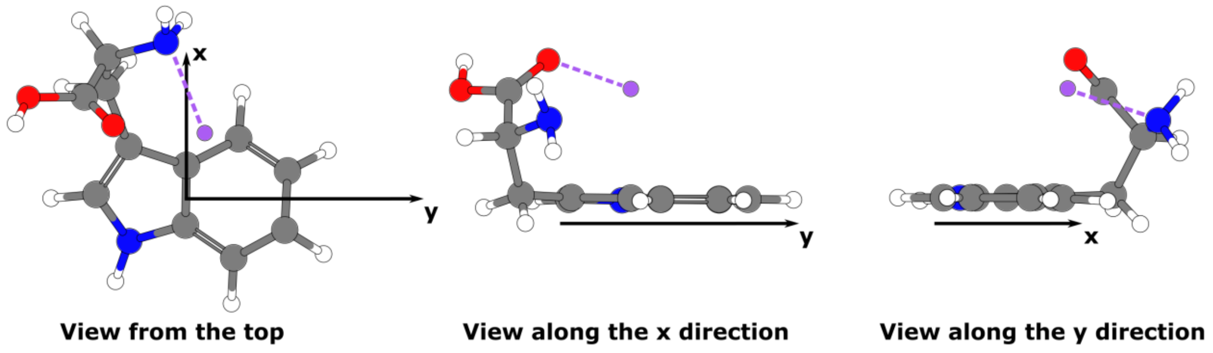

Supplementary Figure 5. Ground state geometry of $TrpNa^+$ under different angles.

3. Excitation Energies of Tryptophan Derivatives

Excited states calculations of the different molecules were performed using RICC2 at the aug-cc-pVDZ level. Supplementary Table 1 shows the excitation energies obtained for $TrpH^+$. The two most stable conformers show similar excitation energies and excited state characters. The two lowest states are the $\pi\sigma^*$ and $\pi\pi^*$ states of interest. In the following, we thus consider the most stable conformer of $TrpH^+$ for the interpretation of experimental trends.

Excited State	Conformer 1	Conformer 2	Literature[3]
1	**4.77 eV ($\pi\sigma^*$)**	**4.77 eV ($\pi\sigma^*$)**	**4.90 eV ($\pi\pi^*$)**
2	**4.85 eV ($\pi\pi^*$)**	**4.87 eV ($\pi\pi^*$)**	**5.04 eV ($\pi\sigma^*$)**
3	**5.00 eV ($\pi\pi^*$)**	**5.04 eV ($\pi\pi^*$)**	**5.23 eV ($\pi\sigma^*$/ $\pi\pi^*$)**
4	**5.34 eV ($\pi\sigma^*$)**	**5.38 eV ($\pi\sigma^*$)**	-
5	**5.76 eV ($\pi\pi_{CO}^*$)**	**5.79 eV ($\pi\pi_{CO}^*$)**	**5.69 eV ($\pi\pi_{CO}^*$)**

Supplementary Table 1. Electronic excited states in $TrpH^+$ conformers. Calculated excitation energies of $TrpH^+$ in the most stable conformer and conformer 2, together with previously reported calculations[3]. The excited state character is indicated, based on the molecular orbitals involved in the transition (see Figure 3 of the main text for the orbitals).

A comparison with previous calculations from the literature[3] is also shown in Supplementary Table 1. While excitation energies remain the same in our computations, the order of the excited states differs, inverting the $\pi\sigma^*$ and $\pi\pi^*$ states. The order is however known to be strongly dependent on the chosen computation method and molecular geometry[6]. To investigate the discrepancies in the state ordering, we performed excited state calculations on various $TrpH^+$ geometries, i.e., our geometry obtained using DFT methodology and the geometry taken from ref 3, obtained using MP2 calculations. In both geometries, RICC2 calculations of the excited states were performed using either (full) aug-cc-pVDZ basis set, as employed in the present work, or aug-cc-pVDZ(N,O)+def2-SV(P)(C,H), used in ref 3. The results are shown in Supplementary Table 2. As shown, both geometries lead to similar excited states. Especially, calculations using the smaller aug-cc-pVDZ(N,O)+def2-SV(P)(C,H) basis set give the same energies and states as in ref 3. The difference between the present calculations and the results in ref 3

therefore comes from the larger basis set used in the present work, which is more suitable to describe the excited states involved in the process. Indeed, the present large basis set takes into account a larger number of diffuse orbitals for C, N and H, that are important in the description of the πσ* states. Further basis set studies would be interesting to understand the precise πσ*-ππ* ordering in comparison with experimental measurements.

Excited State	DFT geometry aug-cc-pVDZ	MP2 geometry aug-cc-pVDZ	DFT geometry aug-cc-pVDZ-SV(P)	MP2 geometry aug-cc-pVDZ-SV(P)
1	**4.77 eV (πσ*)**	**4.75 eV (πσ*)**	**4.97 eV (ππ*)**	**4.90 eV (ππ*)**
2	**4.85 eV (ππ*)**	**4.80 eV (ππ*)**	**5.10 eV (πσ*)**	**5.04 eV (πσ*)**
3	**5.00 eV (ππ*)**	**4.91 eV (ππ*)**	**5.31 eV (πσ*/ ππ*)**	**5.23 eV (πσ*/ ππ*)**
4	**5.34 eV (πσ*)**	**5.35 eV (πσ*)**	**5.76 eV (πσ*)**	**5.70 eV (πσ*/ ππ*)**

Supplementary Table 2. Comparison of different computational methods in TrpH$^+$. Calculated excitation energies of TrpH$^+$ in the DFT geometry used in this work and in the MP2 geometry taken from ref 3, using either aug-cc-pVDZ or aug-cc-pVDZ(N,O)+def2-SV(P)(C,H) basis set.

The result of excited state calculations in the three molecules considered in this work is given in Supplementary Table 3, and corresponds to the excited states displayed in Figure 3 of the main text. In the case of TrpNa$^+$ and AlaTrpNa$^+$, no $\pi\pi_{CO}$* state below 6 eV was found. $\pi\pi_{CO}$* states are however known to be mostly inactive in the ultrafast dynamics following ππ* excitation[6], as confirmed by their high energy in our calculations. They can however play a role in the subsequent dissociation pathways leading to C_α-C_β cleavage[5].

Additionally, the CC2 calculations are compared with lower-level TDDFT/CAM-B3LYP computations at the 6-311+G(d,p) level, for the dipeptide AlaTrpNa$^+$ (last column in Supplementary Table 3). As can be seen, TDDFT calculations give excitation energies and state characters that are close to the CC2 calculations, despite the fact that failures of this methodology are known in the case of charge-transfer states[7], as is the case here.

Excited State	TrpH$^+$	TrpNa$^+$	AlaTrpNa$^+$	AlaTrpNa$^+$ (TDDFT)
1	**4.77 eV (πσ*)**	**4.83 eV (ππ*)**	**4.70 eV (ππ*)**	**4.82 eV (ππ*)**
2	**4.85 eV (ππ*)**	**4.87 eV (ππ*)**	**4.88 eV (ππ*)**	**5.01 eV (ππ*)**
3	**5.00 eV (ππ*)**	**5.06 eV (πσ*)**	**5.16 eV (πσ*)**	**5.40 eV (πσ*)**
4	**5.34 eV (πσ*)**	**5.58 eV (πσ*)**	**5.71 eV (πσ*)**	-
5	**5.76 eV ($\pi\pi_{CO}$*)**	-	-	-

Supplementary Table 3. Electronic excited states in tryptophan-containing species. Calculated excitation energies of TrpH$^+$, TrpNa$^+$, and AlaTrpNa$^+$, together with their character.

4. Potential Energy Surface Calculations of Tryptophan Derivatives

Figure 4 of the main text displays the analysis of the potential energy surfaces (PESs) implied in the relaxation for $TrpNa^+$. The analysis was focused on the PESs involving stretching of the N-X bonds, because of their known implication in ππ*-πσ* dynamics[8]. A similar analysis can be done for $TrpH^+$ and $AlaTrpNa^+$. Supplementary Figure 6 shows the PESs for $TrpH^+$ along the $N\text{-}H_{free}$ and $N\text{-}H_{ind}$ modes. In agreement with previously reported results[2], the πσ* is dissociative along the $N\text{-}H_{free}$ coordinate, being thus responsible for the population transfer, following UV excitation of the ππ* states. While the $N\text{-}H_{ind}$ mode for $TrpH^+$ is equivalent to the N-Na mode in $TrpNa^+$, the πσ* state for $TrpH^+$ is not directly dissociative along this coordinate, i.e., it exhibits a dissociation barrier (that is >0.5 eV in our calculations). It is therefore not implied in the coupling, but it is for instance at play in the excited state proton transfer mechanism where other calculations showed a proton transfer from NH_3^+ to the indole ring[3]. This is in contrast with $TrpNa^+$, highlighting the importance of the position and nature of the bridge atom in the ππ*-πσ* dynamics. Nevertheless, the overall mechanism is very similar, implying the same states coupled by a dissociative N-X coordinate.

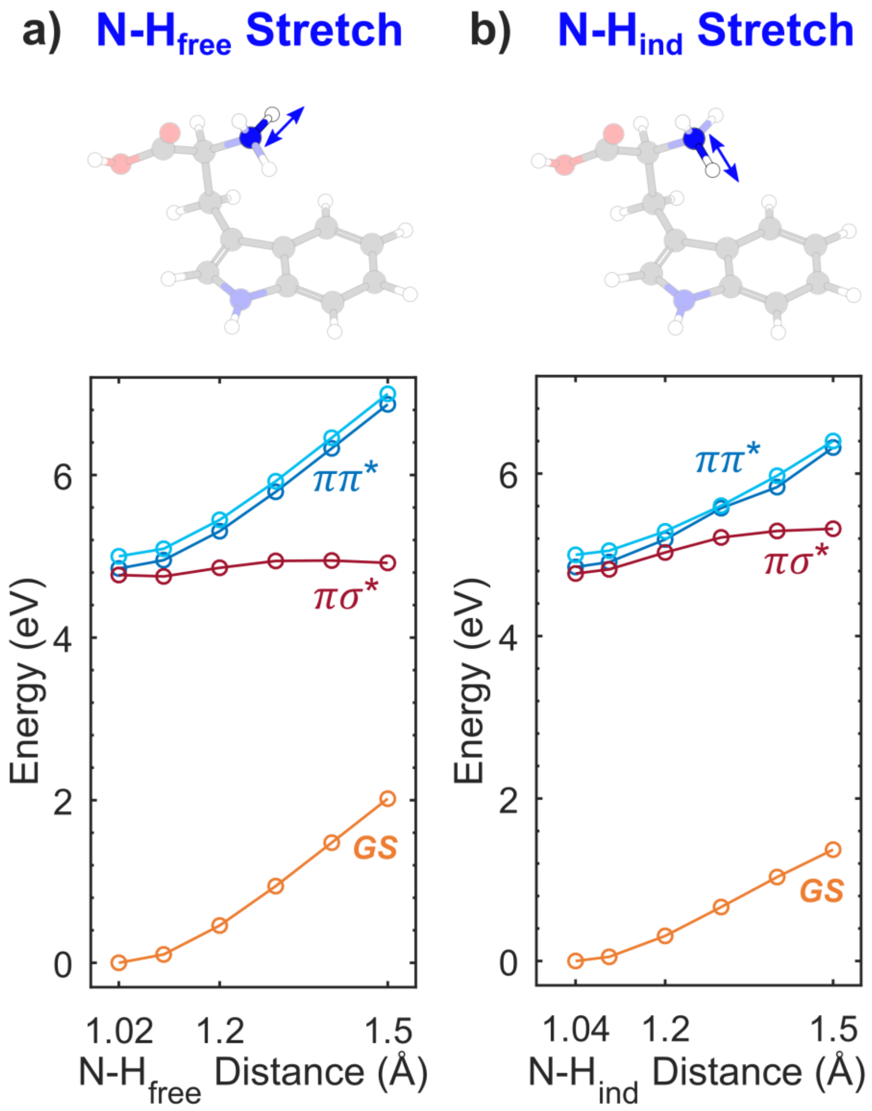

Supplementary Figure 6. Potential energy surfaces of TrpH⁺. Potential Energy Surfaces for TrpH⁺ along the N-H$_{free}$ (a) and N-H$_{ind}$ (b) stretching modes, as depicted by the geometries.

In the case of AlaTrpNa⁺, a behavior similar to TrpNa⁺ is observed, as demonstrated in Supplementary Figure 7. Along the N-H coordinates, both ππ* and πσ* states are bound states, implying that these coordinates are not involved in the non-adiabatic coupling. For AlaTrpNa⁺, the N-Na bond is directed towards the indole group, due to the higher flexibility of the system compared to TrpNa⁺. We thus looked at the PES when moving the Na atom off the NH_2 moiety while keeping its translation parallel to the indole ring, to reveal its dissociative character. Supplementary Figure 7b shows that a small dissociation barrier is present for AlaTrpNa⁺, and crossing between the ππ* and πσ* states occurs again through this coordinate.

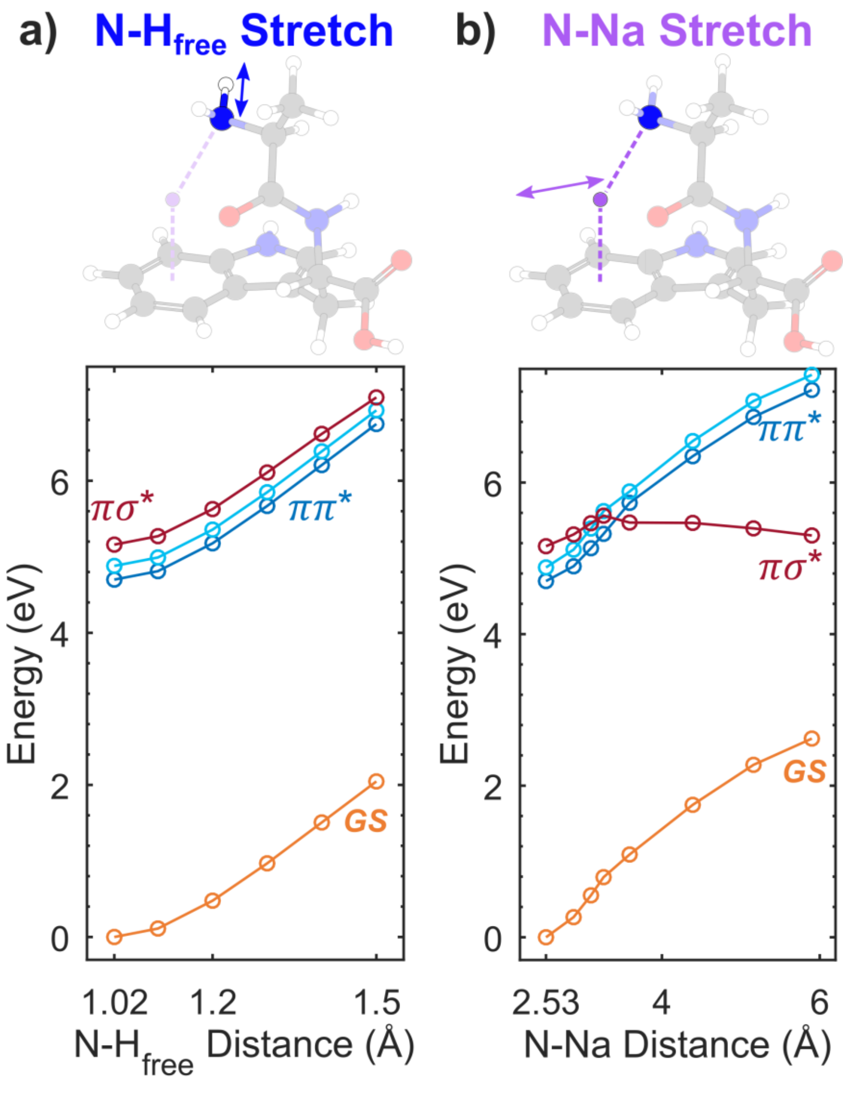

Supplementary Figure 7. Potential energy surfaces of AlaTrpNa⁺. Potential Energy Surfaces for AlaTrpNa⁺ along the N-H$_{free}$ stretching mode (a) and N-Na distance, while maintaining the Na atom parallel to the indole group (b), as depicted by the geometries.

PESs were also computed using TDDFT for AlaTrpNa⁺, and the result is shown in Supplementary Figure 8. Again, the curves derived from the TDDFT calculations are very similar to the CC2 curves in Supplementary Figure 7, and only differ by small shifts in energy. The reasonable results obtained with

TDDFT thus suggest that it could be further used for larger molecular systems (e.g., polypeptides), where higher-hierarchy calculations such as CC2 are less affordable.

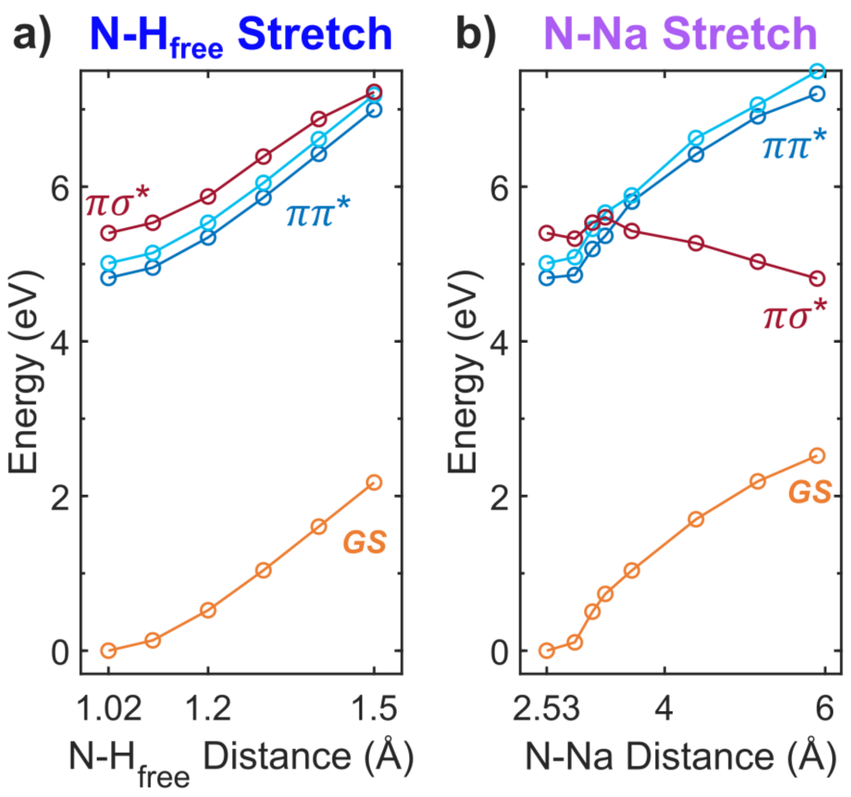

Supplementary Figure 8. Potential energy surfaces of AlaTrpNa$^+$ using DFT calculations. Same as Supplementary Figure 7, but with TDDFT calculations.

Supplementary Discussion

1. Global-Local Stabilization Separation (GLoSS) Model

In order to understand the main parameters responsible for the control of the ππ*-πσ* dynamics, we developed a simple model including first principle arguments to rationalize the observed trend. It is based on the difference of electron density localization between the two ππ* and πσ* electronic states. Thus the relative energy of these two states is changed differently when changing the global or local environment, which is reflected in the specific case of this work by changes in the adduct atom or the peptide chain.

Supplementary Figure 9 illustrates the relative stabilization of the involved ππ* and πσ* states. In the following, the ππ* state is the initially excited state, as confirmed by the oscillator strengths in the calculations, and the πσ* state is the final state, once the population transfer is complete. Let us first consider the case of the ground state (GS) of neutral tryptophan. For neutral tryptophan, the energy gained by addition of an X$^+$ adduct atom corresponds to the X$^+$ affinity, named XA. As the electron density remains localized on the indole group for both GS and the ππ* state, the ππ* state is assumed to be also stabilized by XA from Trp to TrpX$^+$. This means that the ππ* transition energy is assumed to remain the same for Trp and TrpX$^+$. For the πσ* state of TrpX$^+$, the "virtual" equivalent state in neutral Trp can be described by an electron density localized on the NH_2 group, which becomes negatively charged (see the electron density in Supplementary Figure 9). Thus, as a first approximation, attachment of the X$^+$ adduct stabilizes the πσ* state by a long range Coulomb interaction between the added X$^+$ (with a +1 charge) and the NH_2^- moiety of neutral tryptophan (with a -1 charge). In fact, the exact

interaction between the amino group and the adduct atom clearly displays a covalent character, as indicated by the calculated partial charges presented in Supplementary Table 4: for $TrpH^+$, the charge is decreased on the X^+ (+0.49) and N (-0.80) atoms due to covalent exchange of electrons when attaching a proton to a neutral tryptophan molecule. However, this character is not included in the crude model presented here (both X and N atoms keep a charge of ±1). This means that in a first approximation, we consider only the main energy contribution for the πσ* state, that is electrostatic stabilization. Interaction with the positive hole on the indole is also neglected, due to the dilution of the charge on the full ring and larger distances with the adduct.

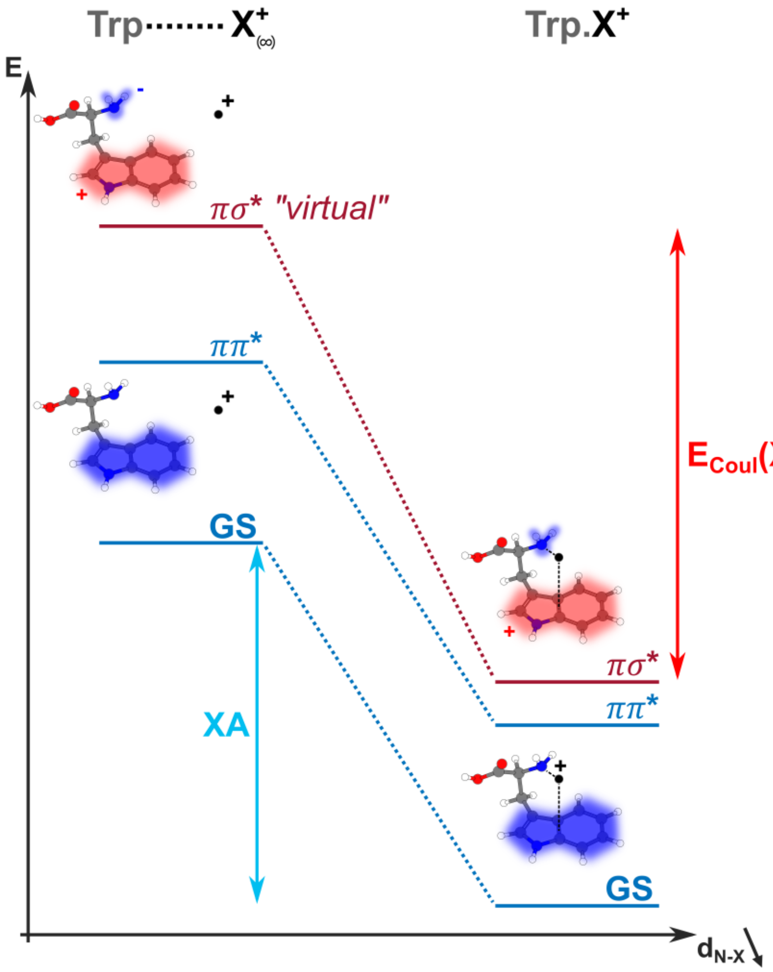

Supplementary Figure 9. Global-local stabilization separation model. Energy diagram of the Global-Local Stabilization Separation model. The excited states of neutral Trp are shown on the left, where the electron density is shown in blue (positively charged hole density in red). When an X^+ charge is bound to the molecule, the energy of the different states changes (right), according to the X^+ Affinity (XA) for the ground state (GS) and the ππ* state, and to Coulomb interaction (E_{Coul}) for the πσ* state.

Thus, in a simple picture, the ππ* state in any $TrpX^+$ molecule is stabilized by global effects on the indole chromophore while the πσ* state energy is mainly impacted by local effects around the NH_2 group, i.e., mainly by the N-X distance R_{NX} via Coulomb forces. As a consequence of these separated contributions, the relative energy gap $\varepsilon_g = E_{\pi\sigma^*} - E_{\pi\pi^*}$ between the two states will vary when changing the X^+ adduct according to:

$$\delta\varepsilon_g(X_1 \rightarrow X_2) = \varepsilon_g(X_2) - \varepsilon_g(X_1) = \Delta XA - \Delta E_{Coul}$$
$$= [XA(X_2) - XA(X_1)] - \frac{e^2}{4\pi\varepsilon_0}\left[\frac{1}{R_{NX_2}} - \frac{1}{R_{NX_1}}\right] \qquad (1)$$

Where $\delta\varepsilon_g(X_1 \rightarrow X_2)$ is the increase in the energy gap between the ππ* and the πσ* states when changing the adduct from X_1^+ to X_2^+. It can be globally generalized to the environment of the molecule, X_1 and X_2 being in this case replaced by two different environments of the indole ring. The GLoSS model thus enables a direct understanding of the evolution of the ππ*-πσ* energy gap. The ππ*-πσ* timescale is then directly linked to this energy gap, since the non-adiabatic coupling between two states is usually inversely proportional to the gap.

Molecule	Charge on indole ring (Ind-CH_2-)	Charge on glycine (-CHNH2COOH)	Charge on N	Charge on X
**$TrpH^+$**	**0.10**	**0.39**	**-0.80**	**0.49**
**$TrpNa^+$**	**0.06**	**-0.03**	**-0.96**	**0.97**

Supplementary Table 4. Partial charges in $TrpH^+$ and $TrpNa^+$. Calculated partial charges (using Natural Population Analysis, NPA) for protonated and sodiated tryptophan, on the indole ring, the glycine moiety, the N atom of the N-terminus and the X adduct.

Supplementary Table 5 gives the evolution of the ππ*-πσ* energy gap obtained from RICC2 calculations. We used the proton and sodium affinities from the literature[9,10] and the N-X distances extracted from our calculations to evaluate the energy gap differences using the GLoSS model. It can be seen that the evolution of the gap is well reproduced by the model, despite its crude approximations. When changing from H^+ to Na^+, both global (XA) and local (R_{NX}) parameters contribute to the change in energy gap, because of the very different nature of H^+ and Na^+, but the overall prediction remains satisfying. Indeed, the proton affinity is closely related to the interaction of the added proton with the N-terminus, together with the global cation-π interaction[11] with the indole ring, while the sodium affinity describes a more global interaction that includes trident complexation of Na^+ by the NH_2, CO and indole moieties. These differences in the global stabilization are balanced by the change of the local N-X distance. When comparing $TrpNa^+$ and $AlaTrpNa^+$, the N-Na distance remains almost constant. The global affinity for Na^+ will however be increased for the dipeptide AlaTrp, because of its higher flexibility. This change in the affinity is consequently responsible for the observed trend in this case. We can thus explain the increase of the ππ*-πσ* timescale by the increase in energy gap, which is sensitive to the overall interaction of the molecule with the adduct atom. While the GLoSS model is not aimed at replacing advanced quantum chemical computations, it provides instructive information on the physical interactions at play in the process.

Additionally, Supplementary Table 5 gives the position and energy barrier of the crossing point between the πσ* and ππ* states, where the energy barrier is defined with respect to the minimum of the ππ* PES. This crossing point is estimated using the PES of Figure 4b of the main text and Supplementary Figure 7b. As observed in Supplementary Table 5, the increase of the vertical energy gap between the two states correlates with an increase of the energy barrier involved in the dynamics, confirming the vertical energy gap as an equivalent criterion in the case of the present dynamics.

Molecule	XA (kJ.mol^{-1})	R_{NX} (Å)	ε_g (X) *ab initio*	$\delta\varepsilon_g$(X) *ab initio*	$\delta\varepsilon_g$(X) GLoSS	Crossing energy
**TrpH$^+$**	**949**	**1.04**	**-80 meV**	**-**	**-**	**-**
**TrpNa$^+$**	**210**	**2.47**	**230 meV**	**310 meV** (TrpH$^+$→TrpNa$^+$)	**395 meV** (TrpH$^+$→TrpNa$^+$)	**100 meV (2.76 Å)**
**AlaTrpNa$^+$**	**228**	**2.53**	**460 meV**	**230 meV** (TrpNa$^+$→AlaTrpNa$^+$)	**326 meV** (TrpNa$^+$→AlaTrpNa$^+$)	**820 meV (3.43 Å)**

Supplementary Table 5. Comparison of calculations with the GLoSS model. Evolution of the πσ*-ππ* energy gap obtained from the *ab initio* (TDDFT/CAM-B3LYP) computations, taking the energy of the lowest of the two ππ* states for $E_{\pi\pi^*}$. The relative variation of the energy gap between TrpH$^+$ and TrpNa$^+$, and between TrpNa$^+$ and AlaTrpNa$^+$ is given, calculated from the *ab initio* computations and from the GLoSS model. The affinities (ref 9,10) and equilibrium distances taken for the model are given in the two first columns. The crossing energy, with respect to the minimum of the PES of the lowest ππ* state, is also given together with its N-Na distance, taken as the energy and N-Na bond length at the crossing point between the πσ* and ππ* states on Figure 4b (main text) and Supplementary Figure 7b.

Finally, the influence of the global complexation of the adduct is confirmed by measurements on another sodiated dipeptide, GlyTrpNa$^+$. Time-resolved measurements, presented in Supplementary Figure 10, reveal a timescale of 23±12 ps for the ππ*-πσ* dynamics in this dipeptide. This value is again increased compared to TrpNa$^+$, and is due to the higher affinity of the dipeptide compared to bare tryptophan, revealing the strength and possibilities brought by the model. The model is thus general to any system with an indole chromophore, maintained in the vicinity of an NH_2 moiety via a bridge X$^+$, thanks to cation-pi interactions[12]. The modulation of the bridge complexation, globally and locally, enables fine tuning of ππ*-πσ* couplings. This control strategy, based on the separation of the contributions due to the micro-environment, can also be extended to any encountered ππ*-πσ* or even charge transfer process, as long as a separated influence on each state can be achieved.

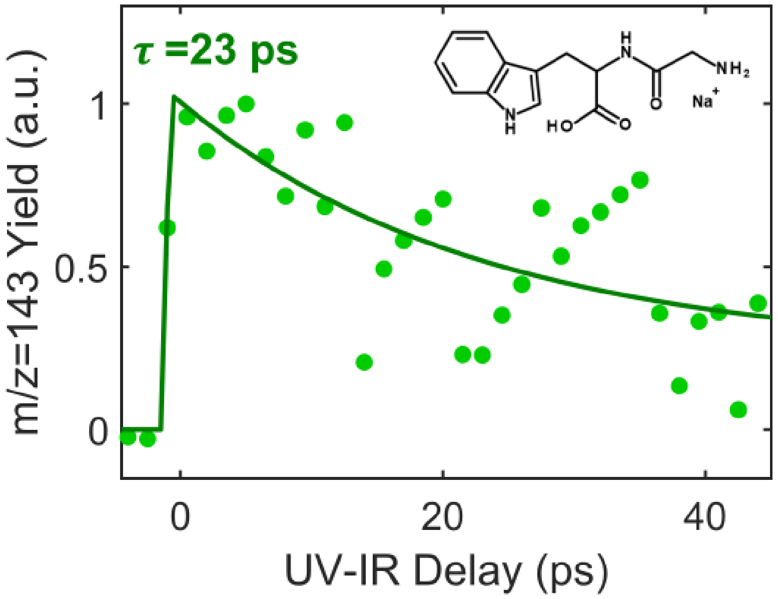

Supplementary Figure 10. Time-resolved mass spectrometry measurements in GlyTrpNa$^+$. Normalized time-dependent yield of fragment m/z=143 of GlyTrpNa$^+$, as a function of the UV-IR delay, together with its exponential fit, giving a decay of 23±12 ps.

N	1.251220	-1.707725	0.649961
H	0.308025	-1.279388	0.692938
C	2.086657	-0.984299	-0.386444
H	2.733907	-1.722524	-0.867282
C	2.989391	-0.052160	0.431174
O	3.033316	-0.098215	1.636306
C	1.188714	-0.278745	-1.435376
H	1.839863	0.367619	-2.025816
H	0.804111	-1.039362	-2.121089
C	0.059238	0.484635	-0.812030
C	0.044927	1.810007	-0.438761
H	0.798823	2.571348	-0.569509
C	-1.225817	-0.063810	-0.434416
N	-1.156022	2.110458	0.152473
H	-1.421921	3.024806	0.483759
C	-1.958895	0.988607	0.176697
C	-1.842694	-1.323639	-0.584894
H	-1.347532	-2.135009	-1.111322
C	-3.256950	0.810560	0.660244
H	-3.805185	1.623621	1.121555
C	-3.130532	-1.501172	-0.102367
H	-3.621115	-2.459535	-0.222497
C	-3.826439	-0.446065	0.519392
H	-4.833182	-0.616613	0.880620
H	1.708958	-1.568211	1.567781
H	1.147316	-2.704463	0.456318
O	3.710062	0.745065	-0.344470
H	4.308915	1.280271	0.203496

Supplementary Table 6. Coordinates of the equilibrium geometry of $TrpH^+$.

N	-1.6967630000	-1.9314060000	0.4914230000
H	-2.4115610000	-2.5667620000	0.1412170000
C	-2.2985710000	-0.6095050000	0.7617230000
H	-3.2481930000	-0.6872480000	1.3015990000
C	-2.5790170000	0.0686430000	-0.5728130000
O	-2.0034310000	-0.1972940000	-1.6119460000
C	-1.3465440000	0.2830250000	1.6195930000
H	-1.9388670000	1.1224540000	1.9895070000
H	-1.0700590000	-0.3039470000	2.5025300000
C	-0.1255710000	0.8122450000	0.9193450000
C	0.0156390000	2.0996140000	0.4562590000
H	-0.6673510000	2.9319130000	0.5360720000
C	1.1038700000	0.1273080000	0.5807330000
N	1.2321490000	2.2519860000	-0.1655990000
H	1.5800510000	3.1150530000	-0.5530640000
C	1.9250080000	1.0634840000	-0.1124160000
C	1.6196860000	-1.1677290000	0.8118480000
H	1.0648030000	-1.8921270000	1.3975910000
C	3.1937490000	0.7349680000	-0.5999580000
H	3.8023420000	1.4646260000	-1.1216190000
C	2.8789910000	-1.4954030000	0.3239670000
H	3.2867910000	-2.4810010000	0.5155610000
C	3.6551800000	-0.5546490000	-0.3829400000
H	4.6363560000	-0.8382760000	-0.7434150000
H	-1.3837890000	-2.3357960000	1.3702530000
O	-3.5072080000	1.0144140000	-0.4749940000
H	-3.6306550000	1.4349900000	-1.3421380000
Na	-0.0851800000	-1.4469930000	-1.3101270000

Supplementary Table 7. Coordinates of the equilibrium geometry of $TrpNa^+$.

N	0.396167	3.307039	0.249232
C	-0.906717	2.670704	0.568813
H	-0.888238	2.424020	1.634393
C	-0.988965	1.353871	-0.218515
O	-0.301294	1.186559	-1.231915
C	-2.121080	3.565923	0.274632
H	-3.059639	3.076602	0.545660
H	-2.051180	4.495765	0.843405
H	-2.167220	3.812236	-0.790043
H	0.649457	3.936360	1.006110
H	0.277980	3.901419	-0.569703
N	-1.809186	0.402706	0.260298
H	-2.435519	0.601305	1.030054
C	-1.903056	-0.918498	-0.340667
H	-1.545217	-0.836991	-1.366447
C	-3.368371	-1.334599	-0.346450
O	-4.216589	-0.817398	0.335462
C	-1.065616	-2.004024	0.423474
H	-1.619019	-2.302607	1.318777
H	-1.020529	-2.885720	-0.221749
C	0.306626	-1.565688	0.843612
C	0.703959	-1.370020	2.142468
H	0.146820	-1.516059	3.055656
C	1.455664	-1.260432	0.023646
N	2.013236	-0.936471	2.186303
H	2.550018	-0.801895	3.028519
C	2.503748	-0.856137	0.902316
C	1.709640	-1.286842	-1.360088
H	0.945261	-1.607859	-2.057293
C	3.757292	-0.443032	0.433484
H	4.546730	-0.146697	1.115116
C	2.956243	-0.889123	-1.830496
H	3.165113	-0.926308	-2.893246
C	3.969628	-0.462261	-0.942547
H	4.938298	-0.177274	-1.336164
O	-3.578809	-2.374968	-1.165378
H	-4.512556	-2.634387	-1.104918
Na	1.872499	1.547508	-0.823309

Supplementary Table 8. Coordinates of the equilibrium geometry of AlaTrpNa^{+}.

Supplementary References

[1] Mino, W. K.; Gulyuz, K.; Wang, D.; Stedwell, C. N.; Polfer, N. Gas-Phase Structure and Dissociation Chemistry of Protonated Tryptophan Elucidated by Infrared Multiple-Photon Dissociation Spectroscopy. *J. Phys. Chem. Lett.* **2011**, *2*, 4, 299-304.

[2] Kang, H.; Dedonder-Lardeux, C.; Jouvet, C.; Grégoire, G.; Desfrançois, C.; Schermann, J.-P.; Barat, M.; Fayeton, J. A. Control of Bond-Cleaving Reactions of Free Protonated Tryptophan Ion by Femtosecond Laser Pulses. *J. Phys. Chem. A* **2005**, *109* (11), 2417-2420.

[3] Grégoire, G.; Jouvet, C.; Dedonder, C.; Sobolewski, A. L. *Ab initio* Study of the Excited-State Deactivation Pathways of Protonated Tryptophan and Tyrosine. *J. Am. Chem. Soc.* **2007**, *129*, 6223-6231.

[4] Kang, H.; Jouvet, C.; Dedonder-Lardeux, C.; Martrenchard, S.; Grégoire, G.; Desfrançois, C.; Schermann, J.-P.; Barat, M.; Fayeton, J. A. Ultrafast Deactivation Mechanisms of Protonated Aromatic Amino Acids Following UV Excitation. *Phys. Chem. Chem. Phys.* **2005**, *7*, 394-398.

[5] Lucas, B.; Barat, M.; Fayeton, J. A.; Perot, M.; Jouvet, C.; Grégoire, G.; Brøndsted Nielsen, S. Mechanisms of Photoinduced Cα-Cβ Bond Breakage in Protonated Aromatic Amino Acids. J. Chem. Phys. **2008**, 128, 164302.

[6] Grégoire, G.; Jouvet, C.; Dedonder, C.; Sobolewski, A. L. On the Role of Dissociative πσ* States in the Photochemistry of Protonated Tryptamine and Tryptophan: An *ab initio* Study. *Chem. Phys.* **2006**, *324*, 398-404.

[7] Dreuw, A.; Head-Gordon, M. Failure of Time-Dependent Density Functional Theory for Long-Range Charge-Transfer Excited States: The Zincbacteriochlorin−Bacteriochlorin and Bacteriochlorophyll−Spheroidene Complexes. *J. Am. Chem. Soc.* **2004**, *126* (12), 4007-4016.

[8] Ashfold, M. N. R.; King, G. A.; Murdock, D.; Nix, M. G. D.; Oliver T. A. A.; Sage, A. G. πσ* Excited States in Molecular Photochemistry. *Phys. Chem. Chem. Phys.* **2010**, *12*, 1218-1238.

[9] Kish, M. M.; Ohanession, G.; Wesdemiotis, C. The Na+ Affinities of α-amino Acids: Side-chain Substituent Effects. *Int. J. Mass Spectrom.* **2003**, *227*, 509-524.

[10] Wang, P.; Wesdemiotis C.; Kapota, C.; Ohanessian, G. The Sodium Ion Affinities of Simple Di-, Tri-, and Tetrapeptides. *J. Am. Soc. Mass Spectrom.* **2007**, *18*, 541-552.

[11] Dougherty, D. A. The Cation-π Interaction. *Acc. Chem. Res.* **2013**, *46* (4), 885-893.
